# Association between BNT162b2 vaccination and reported incidence of post-COVID-19 symptoms: cross-sectional study 2020-21, Israel

**DOI:** 10.1038/s41541-022-00526-5

**Published:** 2022-08-26

**Authors:** Paul Kuodi, Yanay Gorelik, Hiba Zayyad, Ofir Wertheim, Karine Beiruti Wiegler, Kamal Abu Jabal, Amiel A. Dror, Saleh Nazzal, Daniel Glikman, Michael Edelstein

**Affiliations:** 1 Azrieli Faculty of Medicine, Bar-Ilan University, Safed, Israel; 2 Baruch Padeh Medical Centre, Poriya, Israel; 3 Ziv Medical Centre, Safed, Israel; 4 Galilee Medical Centre, Nahariyah, Israel

**Keywords:** Fatigue, Viral infection

## Abstract

The effectiveness of Coronavirus disease 2019 (COVID-19) vaccines against the long-term COVID-19 symptoms expressed by a substantial proportion of patients is not well understood. We determined whether vaccination with the BNT162b2 mRNA vaccine was associated with incidence of reporting long-term symptoms post-SARS-CoV-2 infection. We invited individuals PCR-tested for SARS-CoV-2 infection at participating hospitals between March 2020 and November 2021 to fill an online questionnaire that included information about demographics, acute COVID-19 episode and symptoms they were currently experiencing. Using binomial regression, we compared vaccinated individuals with those unvaccinated and those uninfected, in terms of post-acute self-reported symptoms. Of the 951 infected, 637(67%) were vaccinated. In the study population, the most prevalent symptoms were: fatigue (22%), headache (20%), weakness of limbs (13%), and persistent muscle pain (10%). After adjusting for age, time from beginning of symptoms to responding to the survey, and baseline symptoms, those who received two vaccine doses were less likely than unvaccinated individuals to report any of these symptoms (fatigue, headache, weakness of limbs, persistent muscle pain) by 62%, 50%, 62%, and 66% respectively, (Risk ratios 0.38, 0.50, 0.38, 0.34, *p* < 0.04 in the listed sequence). Compared to the 2447 included individuals who never reported SARS-CoV-2 infection, double-vaccinated participants were no more likely to report any of the mentioned symptoms. Vaccination with 2+ doses of BNT162b2 was associated with a reduced risk of reporting most of the common post-acute COVID-19 symptoms. Our results suggest that BNT162b2 vaccination may have a protective effect against longer term COVID-19 symptoms.

## Introduction

Long coronavirus disease 2019 (Long COVID), also known as post-COVID-19 syndrome, is an emerging and complex health problem that remains poorly characterised. In October 2021, the World Health Organization (WHO) defined long COVID as “A condition which occurs in individuals with a history of probable or confirmed severe acute respiratory syndrome coronavirus-2 (SARS-CoV-2) infection, usually 3 months from the onset of COVID-19 with symptoms that last for at least two months and cannot be explained by an alternative diagnosis. Common symptoms include fatigue, shortness of breath, and cognitive dysfunction but also others involving the musculo-skeletal, cardiac and central nervous systems, which generally have an impact on everyday functioning”^1^. Symptoms of long COVID may fluctuate or relapse over time. Patients purported to have long COVID have reported a wide range of symptoms, but the most prevalent symptoms include fatigue (approximately 58%), shortness of breath (24%), joint pain (19%), chest pain (16%)^2,3^, headache (44%), palpitations (11%), physical limitations, depression (12%), and insomnia (11%)^3,4^. These symptoms may emerge after the initial recovery from an acute COVID-19 episode or be persistent symptoms that do not resolve following the initial COVID-19 illness^5^.

Vaccination against SARS-CoV-2 infection is one of the most important interventions deployed to mitigate the COVID-19 pandemic. The first COVID-19 vaccine campaign with a WHO-approved vaccine started in December 2020, following the authorisation of the Pfizer/BioNTech BNT162b2 mRNA vaccine by the Food and Drug Administration (FDA) for emergency use^6^. In August 2021, the FDA approved the Pfizer/BioNTech vaccine as the first vaccine for use to prevent COVID-19 in individuals sixteen years and older^7^. As of October 2021, the WHO has listed the Pfizer/BioNTech, AstraZeneca-SK Bio, Sinopharm, Serum Institute of India, Janssen, and Moderna vaccines for emergency use under the Emergency Use Authorisation (EUA)^8^. By January 2022, over 58% of the world population had received at least one dose of the EUA COVID-19 vaccines, accounting for 9.2 billion COVID-19 doses^6^. Available evidence demonstrates that COVID-19 vaccines are effective in preventing severe complications of COVID-19 and death^9^, including in cases of infection with the Delta and Omicron variants of SARS-CoV-2^10,11^, variants that are more contagious than the ancestral SARS-CoV-2 variants^12,13^.

In Israel, the COVID-19 vaccination campaign started in December 2020, and by January 2022, approximately 63.6% of the entire population had received at least two doses, almost all with the BNT162b2 mRNA vaccine^14^ and 45.6% had received a third dose, available in Israel from June 2021. The third dose demonstrated high effectiveness against severe outcomes in the context of waning immunity 6 months post-priming course^15^. During the study period, individuals previously infected with SARS-CoV-2 were eligible for a single dose of the BNT162b2 mRNA vaccine only. All other individuals with no history of SARS-CoV-2 infection were eligible for a first and a second dose with eligibility for the second dose at three weeks from the date of the first dose. A third booster dose of the of the BNT162b2 messenger RNA vaccine was approved on 30 June 2021 for individuals 60 years and older who have received two doses of COVID-19 vaccine at least five months earlier^14^.

Risk factors for developing long COVID have not been fully explored. So far, increasing age, pre-existing health conditions such as hypertension, obesity, psychiatric disorders, and immunosuppression have been associated with an increased risk of long COVID^16^. As the process to establish a more specific case definition of long COVID continues, little is known about the impact of COVID-19 vaccination on long COVID. A large prospective study reported an association between vaccination and lower self-reporting of symptoms beyond 28 days among SARS-CoV-2 infected individuals but without reporting details on specific symptoms or duration^17^. Findings from one study suggests vaccination reduces the reporting of some, but not all post-acute COVID-19 sequelae 6 months post-infection^18^. Another study, albeit with a small sample and without a control group, reported a high proportion of COVID-19 breakthrough infections among patients with long COVID symptoms^19^ (preprint). An additional study reported that 20% of individuals infected post-vaccination developed post-COVID-19 symptoms beyond six weeks, however the investigators did not carry out baseline comparisons^20^ (preprint). In the current study, we aimed to determine whether vaccination with the BNT162b2 mRNA vaccine was associated with reporting specific long-term symptoms post-SARS-CoV-2 infection.

## Results

### Characteristics of the study population

Of 79482 invitations to participate in the study, 3572 (4.5%) individuals over the age of 18 agreed to participate: 2447 who reported no previous SARS-CoV-2 infection and 1125 who did. We excluded 174 infected individuals from the study because they did not report their vaccination status.

Of the 951 infected individuals included in the study, 340 (36%) reported receiving a single vaccine dose and 294 (31%) reported having received at least two vaccine doses (of which 27 received a third dose). Of the 2447 individuals reporting no previous infection 21 (0.9%) received one dose, 1195 (48.8%) received two doses, 744 (30.4%) received three doses, and the rest were unvaccinated (19.9%). Of the 951 infected individuals, 636 (67%) reported at least one symptom during COVID-19 diagnosis. Among the unvaccinated, 69% reported at least one symptom at COVID-19 diagnosis, vs. 57% among those who received two doses and 74% among those who received one dose only, (*p* < 0.05, Table 1).

**Table 1:** Baseline characteristics of the study population.

		SARS-CoV-2 infected participants			
Variable^a^	Uninfected participants (*n* = 2447)	All infected participants (*n* = 951)	Received one vaccine dose (*n* = 340)	Received two vaccine doses (*n* = 294)	Unvaccinated (*n* = 317)
Age					
19–35	440 (18.0%)	288 (30.3%)	109 (32.1%)	59 (20.1%)	120 (37.9%)
36–60	981 (40.1%)	468 (49.2%)	171 (50.3%)	135 (45.9%)	162 (51.1%)
>60	703 (28.7%)	195 (20.5%)	60 (17.6%)	100 (34.0%)	35 (11.0%)
Missing	323 (13.2%)	0 (0.0%)	0 (0.0%)	0 (0.0%)	0 (0.0%)
Sex					
Male	723 (29.5%)	283 (29.8%)	962 (28.2%)	100 (34.0%)	87 (27.5%)
Female	954 (39.0%)	467 (49.1%)	175 (51.5%)	136 (46.3%)	156 (49.2%)
Missing	770 (31.5%)	201 (21.1%)	69 (20.3%)	58 (19.7%)	74 (23.3%)
Ethnic identity					
Jewish	735 (30.0%)	325 (34.2%)	118 (34.7%)	97 (33.0%)	110 (34.7%)
Christian/Muslim Arabs/Druze	193 (7.9%)	144 (15.1%)	60 (17.6%)	35 (11.9%)	49 (15.5%)
Missing	1519 (62.1%)	482 (50.7%)	162 (47.7%)	162 (55.1%)	158 (49.8%)
Residence					
City (Over inhabitants)	143 (5.8%)	218 (22.9%)	76 (22.4%)	64 (21.8%)	78 (24.6%)
Town (up to 20,000 residents)	74 (3.0%)	67 (7.1%)	26 (7.7%)	16 (5.4%)	25 (7.9%)
Community/ Kibbutz^b^	421 (17.2%)	154 (16.2%)	60 (17.6%)	48 (16.3%)	46 (14.5%)
Others	168 (6.9%)	21 (2.2%)	12 (3.5%)	3 (1.0%)	6 (1.9%)
Missing	1641 (67.1%)	491 (51.6%)	166 (48.8%)	163 (55.5%)	162 (51.1%)
Education level					
Tertiary institution	352 (14.4%)	248 (26.1%)	95 (27.9%)	78 (26.5%)	75 (23.7%)
Vocational training	77 (3.1%)	81 (8.5%)	28 (8.2%)	22 (7.5%)	31 (9.8%)
High school	119 (4.9%)	93 (9.8%)	37 (10.9%)	21 (7.2%)	35 (11.0%)
Elementary school	21 (0.9%)	12 (1.3%)	6 (1.8%)	3 (1.0%)	3 (0.9%)
Missing	1878 (76.7%)	517 (54.4%)	174 (51.2%)	170 (57.8%)	173 (54.6%)
Body mass index^c^					
Underweight (<20)	18 (0.7%)	10 (1.0%)	4 (1.2%)	2 (0.7%)	4 (1.3%)
Normal (20–25)	319 (13.0%)	165 (17.4%)	631 (18.5%)	47 (16.0%)	55 (17.3%)
Overweight (>25, <30)	325 (13.3%)	175 (18.4%)	58 (17.1%)	55 (18.7%)	62 (19.6%)
Obese (30+)	248 (10.2%)	108 (11.4%)	49 (14.4%)	27 (9.2%)	32 (10.1%)
Missing	1537 (62.8%)	493 (51.8%)	166 (48.8%)	163 (55.4%)	164 (51.7%)
Pre-SARS-CoV-2 infection Chronic Conditions					
Hypertension	NA	85 (8.9%)	28 (8.2%)	41 (14.0%)	16 (5.1%)
Asthma	NA	34 (3.6%)	8 (2.4%)	11 (3.7%)	15 (4.7%)
Diabetes mellitus	NA	60 (6.3%)	24 (7.1%)	24 (8.2%)	12 (3.8%)
Chronic obstructive pulmonary disease	NA	15 (1.6%)	8 (2.3%)	1 (0.3%)	6 (1.9%)
Missing		757 (79.6%)	272 (80.0%)	217 (73.8%)	268 (84.5%)
Hospitalised	NA	85 (8.9%)	35 (10.3%)	21 (7.1%)	29 (9.1%)
Not hospitalised	NA	866 (91.1%)	305 (89.7%)	273 (92.9%)	288 (90.9%)
COVID-19 symptomatic at SARS-CoV-2 infection	NA	636 (66.9%)	252 (74.1%)	167 (56.8%)	217 (68.5%)
Asymptomatic at SARS-CoV-2 infection	NA	315 (33.1%)	88 (25.9%)	127 (43.2%)	100 (31.5%)
Time from beginning of symptoms to responding to the survey (Days)	NA	302 (296)^d^	348.00(166)^d^	114.50(340)^d^	246.50 (189)^d^

NA—the group of participants were not asked these question.

^a^Not all participants answered all questions, therefore the number of responses vary for each question.

^b^Kibbutz - small collectivist residences.

^c^BMI- according to World Health Organisation classification.

^d^(median, IQR).

Compared with individuals vaccinated with one and two doses, unvaccinated participants were younger (52 and 44 vs. 39 years, respectively, *p* < 0.001, Table 1), reflecting the COVID-19 vaccination patterns in the general population and vaccination policy which made vaccines available to older individuals earlier. Consequently, pre-existing chronic conditions were more frequently reported in the vaccinated group (*p* < 0.05). The vaccinated and unvaccinated groups were comparable in terms of gender distribution and socio-economic characteristics (Table 1). The median time between acute illness and answering the questionnaire was longer in the unvaccinated group compared to those who received two doses (8 vs. 4 months, *p* < 0.05). The median time from beginning of symptoms to responding to the survey was longest among individuals vaccinated with one dose (11 months), likely reflecting the fact that individuals who received one dose were by and large infected before being vaccinated. Out of 951 infected participants, 85 (9%) reported having been hospitalised with comparable proportions in all groups (the vaccinated with one dose, the vaccinated with two doses and the unvaccinated (*p* = 0.27, Table 1). The age distribution and socio-demographic characteristics among those reporting no previous infection was comparable to those who received two doses of vaccine (Table 1).

### Post-COVID-19 symptoms among vaccinated and unvaccinated participants

**Fig. 1:**
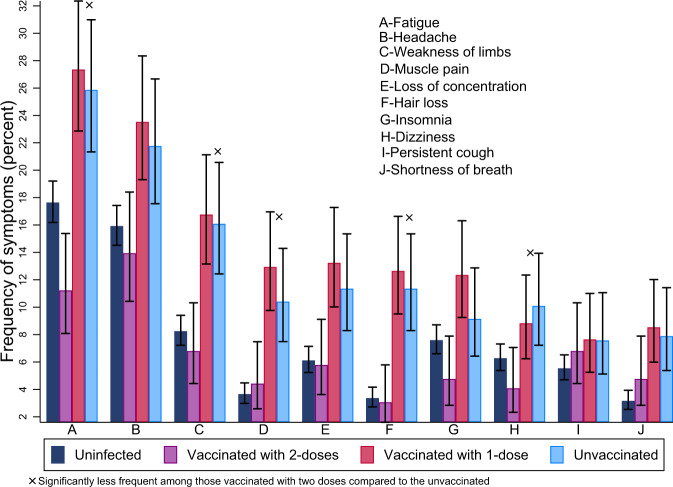
Frequency of most reported symptoms among the uninfected, the infected and unvaccinated, and the infected and vaccinated with 1 or 2 vaccine doses. Error bars represent 95% confidence intervals.

Of the 951 infected participants, 337 (35%) reported not fully recovering from the initial COVID-19 symptoms at follow-up. The most commonly reported symptoms at the time of responding to the survey were fatigue (22%), headache (20%), weakness in arms or legs (13%), and persistent muscle pain (10%) (Table 2 and Fig. 1).

**Table 2:** Post COVID-19 symptoms among vaccinated and unvaccinated participants.

	Number and proportion experiencing post COVID symptoms			
Post COVID symptoms	All participants (*n* = 951)	Received 1 vaccine dose (*n* = 340)	Received two vaccine doses (*n* = 294)	Unvaccinated (*n* = 317)
Fatigue	208 (21.9%)	93 (27.4%)	33 (11.2%)	82 (25.9%)
Headache	190 (20%)	80 (23.5%)	41 (14%)	69 (21.8%)
Weakness in arms or legs	128 (13.5%)	57 (16.8%)	20 (6.1%)	51 (16.1%)
Persistent muscle pain	98 (10.3%)	45 (13.2%)	17 (5.8%)	36 (11.4%)
Loss of concentration	90 (9.5%)	44 (12.9%)	13 (4.4%)	33 (10.4%)
Hair loss	88 (9.3%)	43 (12.7%)	9 (3.1%)	36 (11.4%)
Problem sleeping	85 (8.9%)	42 (12.4%)	14 (4.8%)	29 (9.2%)
Dizziness	74 (7.8%)	30 (8.8%)	12 (4.1%)	32 (10.1%)
Persistent cough	70 (7.4%)	26 (7.7%)	20 (6.8%)	24 (7.6%)
Shortness of breath	68 (7.2%)	29 (8.5%)	14 (4.8%)	25 (7.9%)
Loss of taste	63 (6.6%)	20 (5.9%)	15 (5.1%)	28 (8.8%)
Chest pains	61 (6.4%)	24 (7.1%)	14 (4.8%)	23 (7.3%)
Pins and needles sensation	60 (6.3%)	31 (9.1%)	6 (2%)	23 (7.3%)
Palpitations	57 (6%)	26 (7.7%)	12 (4.1%)	19 (6%)
Depression and anxiety	55 (5.8%)	19 (5.6%)	17 (5.8%)	19 (6%)
Abdominal pain	54 (5.7%)	24 (7.1%)	8 (2.7%)	22 (6.9%)
Problems with balance	52 (5.5%)	24 (7.1%)	7 (2.4%)	21 (6.6%)
Inability to control body movement	49 (5.2%)	22 (6.5%)	9 (3.1%)	18 (5.7%)
Joint pain or swelling	47 (4.9%)	22 (6.5%)	5 (1.7%)	20 (6.3%)
Loss of smell	41 (4.3%)	9 (2.7%)	9 (3.1%)	23 (7.3%)
Loss of appetite	40 (4.2%)	11 (3.2%)	12 (4.1%)	17 (5.4%)
Pain on breathing	40 (4.2%)	16 (4.7%)	8 (2.7%)	16 (5.1%)
Nausea and vomiting	37 (3.9%)	11 (3.2%)	7 (2.3%)	19 (6%)
Constipation	34 (3.6%)	12 (3.5%)	8 (2.7%)	14 (4.4%)
Erectile dysfunction	32 (3.4%)	12 (3.5%)	8 (2.7%)	12 (3.8%)
Diarrhoea	31 (3.3%)	11 (3.2%)	5 (1.7%)	15 (4.7%)
Double vision	29 (3.1%)	17 (5%)	3 (1%)	9 (2.8%)
Problems speaking or communicating	28 (2.9%)	15 (4.4%)	3 (1%)	10 (3.2%)
Weight loss	28 (2.9%)	6 (1.8%)	8 (2.7%)	14 (4.4%)
Tremor/shakiness	27 (2.8%)	11 (3.2%)	8 (2.7%)	8 (2.5%)
Problems passing urine	16 (1.7%)	6 (1.8%)	2 (0.7%)	8 (2.5%)
Loss of sensation, one side of the body	15 (1.6%)	7 (2.1%)	2 (0.7%)	6 (1.9%)
Skin lumps or rashes	13 (1.4%)	5 (1.5%)	2 (0.7%)	6 (1.9%)
Problems swallowing or chewing	12 (1.3%)	5 (1.5%)	3 (1%)	4 (1.3%)
Blood clots in veins	12 (1.3%)	5 (1.5%)	3 (1%)	4 (1.3%)
Kidney problems	12 (1.3%)	5 (1.5%)	2 (0.7%)	5 (1.6%)
Fainting/blackouts	10 (1.1%)	4 (1.2%)	4 (1.4%)	2 (0.6%)
Seizures/fits	10 (1.1%)	4 (1.2%)	3 (1%)	3 (1%)
Heart attack & stroke	3 (0.3%)	2 (0.6%)	1 (0.3%)	0 (0%)

### Risk of reporting post-COVID-19 symptoms according to vaccination status

**Fig. 2:**
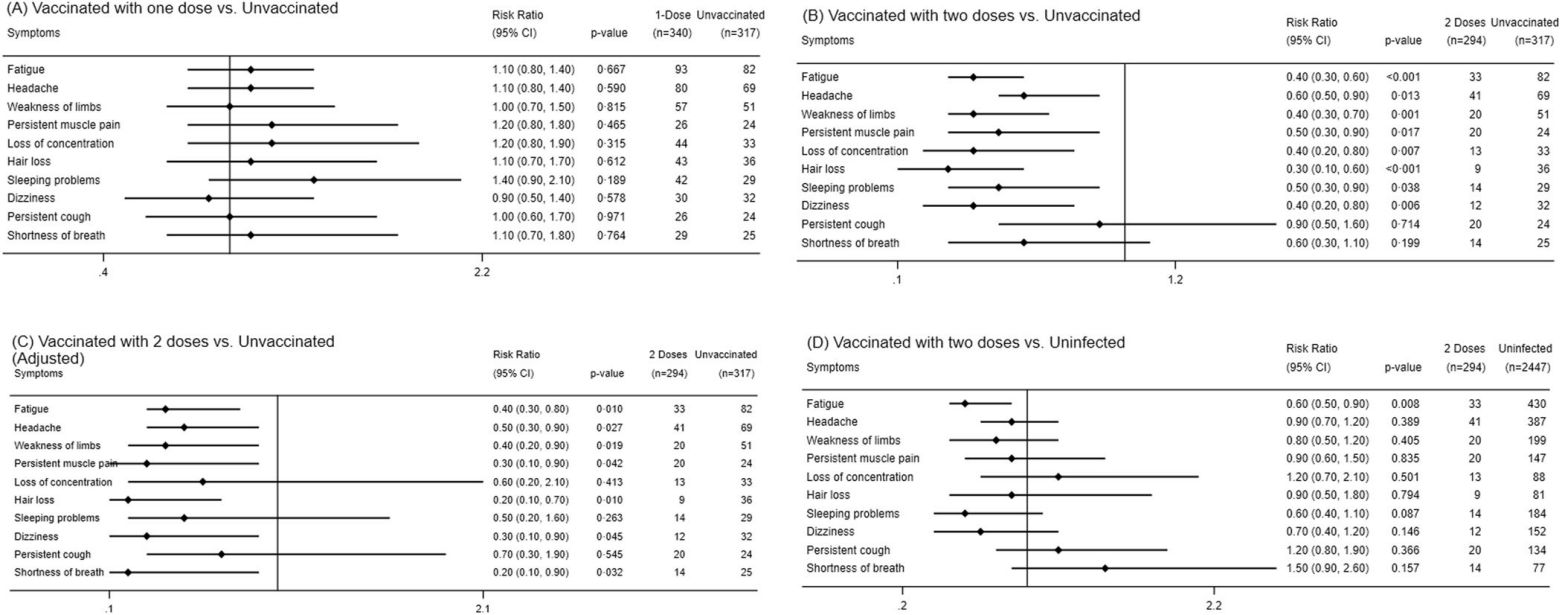
Risk ratios for the ten most frequent post-COVID symptoms. Post-COVID symptoms among one-dose vaccinated participants compared to unvaccinated (**A**), two-dose vaccinated compared to unvaccinated (crude, **B**), two-dose vaccinated compared to unvaccinated (adjusted, **C**) and two-dose vaccinated compared to uninfected (**D**). Error bars represent 95% confidence intervals.

Of the 39 symptoms and diagnoses reported by the patients, 35 (90%) were reported less commonly among those who received at least two doses of vaccine compared with those unvaccinated (Table 2). Most of these symptoms were reported only rarely, in insufficient numbers to include in a meaningful regression analysis. The main regression analysis described thereafter focuses on the ten symptoms most commonly reported in the study, presented in Fig. 2, with the analysis for the rest of the symptoms available in Supplementary Table 2. Compared with unvaccinated participants, those who received two doses were 36–73% less likely to report eight of the ten most commonly reported symptoms (*p* < 0.04 for all, Fig. 2). After adjusting for time from beginning of symptoms to responding to the survey, age and baseline symptoms, a 50–81% reduction in reporting symptoms among those who received two doses for seven of the ten most commonly reported symptoms was detected (Fig. 2). Infected individuals who received two doses of COVID-19 vaccine were no more likely to report any of these symptoms than individuals who reported never having been infected with SARS-CoV-2 (Fig. 2).

In an exploratory age-stratified analysis, differences in reported symptoms were primarily seen in the older age groups, in particular those aged above 60 years (Supplementary Table 1). We emphasise that once stratified, the number of patients in each age group is small and the analysis becomes underpowered; the age-specific results should be interpreted with caution.

## Discussion

Overall, receipt of two doses of the BNT162b2 mRNA vaccine was associated with a substantial decrease in reporting most symptoms post SARS-CoV-2 infection. Individuals who had received two doses reported no more of these symptoms than individuals never reporting infection. Commonly reported post-acute COVID-19 symptoms are not specific to COVID-19 and are commonly reported regardless of infection status, for a variety of reasons. It is not therefore expected that any group would report zero incidence of such symptoms. The absence of difference in symptoms frequency among those who received two doses and those who reported never having been infected, in addition to the difference between those vaccinated and those not, strengthens our findings. These associations were largely not seen among individuals who received a single dose of a COVID-19 vaccine. Our results are consistent with the majority of other available studies on the topic demonstrating lower reporting of long-term symptoms following COVID-19^17,19,21^. A recent review of the few available studies suggests most studies are finding a similar association^22^. The magnitude of the association in this study is on the high end of the spectrum, comparable to some other published studies^17^, but larger than others^21^.

This study brings previously unavailable nuance on the effect of vaccination on reported long-term symptoms, in terms of comparison to baseline incidence of such symptoms, symptom specificity, and time from acute infection. Approximately a third of participants in the current study continued to report post-COVID-19 symptoms, mostly fatigue, headache, and weakness. Our findings are consistent with a recently published study reporting similar proportions of post-COVID-19 symptoms^19^. An exploratory age-stratified analysis showed that the associations reported in the current study were largely confined to older age groups (above 60 years), a finding not consistent with a previous study reporting that the reduced reporting of symptoms was stronger in younger age groups^23^. It is important to note that our age-stratified analysis was underpowered and studies specifically addressing the effect of age should be conducted before definite conclusions are drawn. Our analysis does not allow us to establish why the reduction of long-term symptoms with the COVID-19 vaccine seems to be greater in older age groups. A plausible explanation for the association found in the older individuals could be that younger individuals have more physiological reserve and are therefore able to recover on their own, which is not the case in older adults. Other studies have reported that frailty was associated with both age and worse outcomes following SARS-CoV-2 infection^24^.

The unvaccinated and the vaccinated groups were comparable in socio-demographic characteristics except for age, reflecting vaccine coverage in the Israeli population^25^. As a result, some chronic conditions present at baseline were more common in the vaccinated group. However, since these conditions are more common in the older group who received two doses, we would anticipate even bigger differences between the vaccinated and unvaccinated group had we taken them into account in the analysis. This difference in age between the vaccinated and the unvaccinated individuals is consistent with the COVID-19 vaccination strategy implemented by the Israeli Ministry of Health, which targeted the older population during the initial stages of the COVID-19 vaccination rollout. The vaccinated and unvaccinated populations differed in terms of the proportion of asymptomatic individuals at acute COVID-19 presentation. A higher proportion of those who received two doses were asymptomatic at the time of diagnosis compared to the unvaccinated group, and those who were symptomatic at baseline reported less symptoms compared with those unvaccinated. These figures reflect the possibility of protection against symptomatic disease conferred by vaccines, which may also partly explain the lower proportion of reporting long-term symptoms among those vaccinated. However, the protective effect of vaccination against long-term symptoms persisted after adjusting for asymptomatic disease, suggesting a reduction in reported post-SARS-CoV-2 infection symptoms even among those symptomatic at the time of infection. The median follow-up duration was longer in the unvaccinated group than the individuals vaccinated with two doses. This is consistent with the fact that our follow-up period started nine months before vaccines became available in Israel. Nevertheless, the protective effect of vaccination remained after adjusting for time from beginning of symptoms to responding to the survey. The adjusted model suggested an even stronger protective effect of vaccination against reported long-term symptoms.

Our study relied on self-reported positive PCR results and was conducted during a period when PCR testing was freely and widely available to all. In Israel, most individuals have been PCR-tested multiple times for a range of reasons including being symptomatic, travelling, contact tracing, and screening. Specimens for the same patients are tested in different laboratories. Therefore, using positive lab results from a selection of laboratories (rather than all laboratories in the country) could lead to misclassification. Considering the consequences on daily life, including quarantine, it is unlikely that someone would forget having tested positive for SARS-CoV-2, or vice versa. It is however important to note that we could not completely validate that individuals who reported being never-infected, on the basis of never having tested positive, had an infection that was not detected. However, the fact that all individuals in the uninfected group tested negative at least once (and likely multiple times for most) and did not report any acute COVID-19-like episode mitigates the risk of misclassification. It is also important to note that the vaccination policy in Israel, at the time of the survey, specified that SARS-CoV-2-infected individuals were in theory only eligible for a single dose of vaccine. Therefore, individuals who received one dose also differed from those who received two doses in terms of the sequence of events: while those who received two doses have mostly been infected after having been vaccinated, many among those who received a single dose have been infected prior to vaccination. The proportion of individuals eligible for two doses who only received one was small. Although our data does not allow to establish this sequence of events for each individual, the time from beginning of symptoms to responding to the survey reflect this difference. Infection prior to vaccination could partially explain the observed lack of effect of one dose of vaccine regarding long-term reported symptoms. The effect of vaccination on long-term sequelae according to the infection/vaccination sequence warrants further research. With only few patients reporting having been hospitalised, our cohort reflects the mild end of the COVID-19 spectrum, and the results cannot necessarily be extrapolated to patients who were more severely ill (and hospitalised) in the acute phase of the illness. Likewise, it is conceivable that individuals severely affected by their post-SARS-CoV-2 infection symptoms were less likely to reply to the survey, and we cannot ensure that the results are generalisable to this category of individuals. Our study did not include children who are less likely to develop severe acute illness following infection but do report long-term effects^26^. Prevention of long-term symptoms could be one of the most important benefits of vaccination in this age group. At the time of this study, children under 12 years were not eligible for COVID-19 vaccines in Israel. A final limitation is the self-reported nature of the symptoms, in particular since individuals who are eager to vaccinate may differ from those who do not vaccinate in terms of perceptions of health and illness. In addition, asking individuals about a list of symptoms they experience enables to quantify but not qualify their experience. Reported fatigue may translate to very different experiences for different individuals with a very different impact on their daily living. Studies that measure the association between vaccination and long COVID in terms of activities of daily living, beyond symptom categories, are needed. We minimised selection bias in this study by inviting all individuals who tested for SARS-CoV-2 at the study sites to participate. However, recall and selection bias inherent to the methodology of cross-sectional population surveys cannot be entirely ruled out. As a final limitation, Israel used almost exclusively the BNT162b2 mRNA vaccine, and the results are therefore only applicable to that vaccine.

Our findings suggest that in addition to preventing severe disease and death^27,28^, the BNT162b2 mRNA vaccine used in Israel, may be associated with a reduction in the reporting of symptoms post-acute SARS-CoV-2 infection. Despite the methodological limitations described the strength of the effect and the consistency with the existing literature are encouraging. Studies such as this one should be complemented by studies objectively measuring long-term health outcomes in COVID-19 patients in a clinical setting as well as studies investigating plausible biological mechanisms, which may help explain why an association is found with most symptoms, but not all. Our cohort, which continues to recruit participants, will enable us to report with more precision whether the BNT162b2 mRNA’s protective effect reported in this study is sustained, how vaccination impacts on activities of daily living post-COVID, as well as the effect of the different SARS-CoV-2 variants and vaccination on post-COVID symptoms.

## Methods

### Study design and participants

The current study reports results of a cross-sectional study nested in a prospective longitudinal cohort study to assess risk of and risk factors for long-term physical, mental, and psychosocial consequences of COVID-19. All individuals over the age of 18 who were tested for SARS-CoV-2 infection by reverse transcription polymerase chain reaction (RT-PCR) between 15 March 2020 and 15 November 2021 in the three major government hospitals in Northern Israel, namely Ziv Medical Centre, Padeh-Poriya Medical Centre, and Galilee Medical Centre, were eligible to join the study regardless of the test result. Using available patient telephone records, individuals were invited to participate in the study between July 16 and November 18, 2021, through a Short Message Service (SMS) containing an invitation with a link to an online survey available in four commonly spoken languages in Israel: Hebrew, Arabic, Russian, and English. Two reminders to complete the survey were sent to non-responders. In this study, we first restricted the analysis to individuals who reported being infected with SARS-CoV-2 and diagnosed by PCR in order to compare vaccinated to unvaccinated individuals in terms of reported long-term outcomes. Because the most commonly reported long-term outcomes reported by COVID-19 patients are not specific to COVID-19, we then compared the frequency of reported symptoms among infected and vaccinated individuals to never-infected individuals.

### Measurement tools

The data collection tool was modified from the International Severe Acute Respiratory and emerging Infection Consortium (ISARIC) COVID-19 tools developed by a global follow-up working group and informed by a wide range of global stakeholders with expertise in clinical research, outbreak research, infectious disease, epidemiology, respiratory, critical care, rehabilitation, neurology, psychology, rheumatology, cardiology, oncology, and public health medicine^29^. The ISARIC questionnaire was adapted to the Israeli context. Study participants were asked to select from a list all symptoms they were currently experiencing.

### Data collected

The online survey included information about socio-demographic status (place of residence, age, sex, ethnicity/religious affiliation, education level, and income), baseline health status: (Body mass index [BMI] and chronic conditions), information related to the clinical experience and symptoms experienced during the initial COVID-19 diagnosis, SARS-CoV-2 test results, vaccination status, number of doses, type of vaccine and date of administration. Participants were also asked to report symptoms experienced at the time of filling the questionnaire. Explicit consent was sought and collected via the online link, prior to collecting any other information.

### Outcomes

The primary outcome in this study was the proportion, overall, and in specific age groups, of participants reporting selected health outcomes according to vaccination and infection status. It was not possible to determine vaccination status at the time of infection. As a result, some individuals were infected prior to vaccination while others were vaccinated after. Because of vaccination policy in Israel at the time of the study that recommended a single dose for previously infected individuals, it is likely that most individuals who received a single dose were infected prior to vaccination whereas those who received two doses were infected after receiving their vaccines. The complete list of symptoms can be found in Table 2.

### Statistical analysis

We compared the vaccinated and unvaccinated groups’ socio-demographic and health characteristics using Chi-square tests (for proportions) and Student’s *t*-tests (for means). We also compared the proportion of participants presenting with symptomatic disease at baseline using Chi-square tests. Proportions of long-term symptoms and selected health outcomes were calculated for each group with the total number of participants in each group taken as the denominator. We also compared the time elapsed between the onset of COVID-19 symptoms for symptomatic patients and the date of response to the survey in the two groups (vaccinated and unvaccinated).

A first series of binomial regression models were fitted to the data of the participants who reported post-COVID-19 symptoms according to vaccination status. We adjusted for age, the difference in time from beginning of symptoms to responding to the survey and proportion of asymptomatic patients at the time of diagnosis between the groups. We then compared vaccinated and infected individuals to never-infected individuals in terms of reported symptoms, also using binomial regression models. The frequency of most of the 39 symptoms and diagnoses reported was too low to meaningfully include in a regression model, and we therefore focused our main analysis on the ten symptoms most commonly reported in the study, although the frequency according to vaccination status is described for all symptoms in Table 2 and analysed in supplementary table 2. In addition to the age-adjusted analysis, we also conducted an exploratory, age-stratified analysis to determine any age-specific effect. This analysis was however underpowered, and the results should be interpreted accordingly.

Vaccination status was recorded as either one dose or two doses. At the time of data collection, a minority of individuals had received a third dose and those who did were recorded as two doses. The third booster dose of the BNT162b2 messenger RNA vaccine was only introduced in June 2021 and for a specific age group (adults 60 years and older).

Datasets were processed using Microsoft Excel and analysed using STATA version 15.

### Reporting summary

Further information on research design is available in the Nature Research Reporting Summary linked to this article.

## Supplementary information

Supplementary Info

REPORTING SUMMARY

## Data Availability

Minimal data set, supplementary material are published at the Harvard Dataverse via this link: 10.7910/DVN/N5I10C.

## Code availability

Statistical code is published at the Harvard Dataverse via this link: 10.7910/DVN/N5I10C.
