# Supplementary material for: Association between BNT162b2 vaccination and reported incidence of post-COVID-19 symptoms: cross-sectional study 2020-21, Israel
Source: NPJ Vaccines. 2022 Aug 26;7:101. doi: 10.1038/s41541-022-00526-5 (PMC9411827; doi:10.1038/s41541-022-00526-5)
Supplement: Supplementary file 1 — Supplementary Info [file 41541_2022_526_MOESM1_ESM.pdf]

Supplementary table 1. Crude and age-specific risk ratios of the most frequent post COVID symptoms among participants												
			Vaccinated vs unvaccinated (Infected individuals)								Two doses vaccinated + infected vs uninfected	
			Unadjusted analysis						Adjusted analysis*			
**Symptoms and age**	**Uninfected (n=2447)**	**Unvaccinated (n=317)**	**Received one dose (n=340)**			**Received two doses (n=294)**			**Received two doses (n=294)**			
	**n**	**n**	**n**	**RR (95% CI)**	**p-value**	**n**	**RR (95% CI)**	**p-value**	**RR (95% CI)**	**p-value**	**RR (95% CI)**	**p-value**
Fatigue												
19-35	108	29	31	1·2 [0·8–1·8]	0·463	13	0·9 [0·5–1·6]	0·753	0·6 [0·2–1·8]	0·343	0·9 [0·6–1·5]	0·715
36-60	221	46	51	1.1 [0·8–1·5]	0·660	16	0·4 [0·3–0·7]	**0·001**	0·3 [0·1–0·8]	**0·015**	0·5 [0·3–0·9]	**0·008**
>60	98	7	11	0·8 [0·4–1·9]	0·664	4	0·2 [0·1–0·6]	**0·005**	0·2 [0·0–1·3]	0·094	0·3 [0·1–0·8]	**0·012**
Headache												
19-35	131	30	29	1.1 [0·7–1·7]	0·781	14	1·0 [0·6–1·7]	0·853	0·4 [0·1–1·0]	0·060	0·8 [0·5–1·3]	0·381
36-60	192	32	42	1·2 [0·8–1·9]	0·282	24	0·9 [0·6–1·5]	0·660	0.6 [0·3–1·3]	0·206	0·9 [0·6–1·3]	0·624
>60	64	7	9	0·8 [0·3–1·9]	0.590	3	0·2 [0·1–0·6]	**0·007**	0·4 [0·0–4·5]	0·457	0·3 [0·1–1·0]	0·056
Weakness in arms and legs												
19-35	31	18	13	0·8 [0·4–1·5]	0·499	5	0·6 [0·2–1·5]	0·234	0·8 [0·2–3·2]	0·753	1·2 [0·5–3·0]	0·667
36-60	100	26	34	1·3 [0·8–2·1]	0·280	11	0·5 [0·3–1·0]	0·061	0·5 [0·2–1·3]	0·152	0·8 [0·4–1·5]	0·461
>60	68	7	10	0·8 [0·3–1·8]	0·518	4	0·2 [0·1–0·6]	**0·005**	0·1 [0·0–0·5]	**0·008**	0·4 [0·2–1·1]	0·079
Persistent muscle pain												
19-35	31	10	12	1·0 [0·5–2·7]	0·832	6	0·9 [0·3–2·8]	0·862	0·3 [0·0–2·4]	0·247	1·0 [0·4–2·7]	0·962
36-60	70	12	10	1·2 [0·7–2·0]	0·477	9	0·6 [0·3–1·1]	0·109	0·4 [0·1–1·4]	0·146	1·1 [0·6–2·1]	0·670
>60	46	2	4	1·1 [0·3–3·4]	0·921	5	0·2 [0·0–1·0]	0·051	-	-	0·3 [0·1–1·2]	0·097
Loss of concentration												
19-35	30	13	12	1·0 [0·5–2·1]	0·966	5	0·8 [0·3–2·1]	0·624	2·5 [0·2–26·2]	0·457	1·3 [0·5–3·1]	0·617
36-60	39	17	27	1·5 [0·9–2·7]	0·153	6	0·4 [0·2–1·0]	0·061	0·3 [0·3–2·3]	0·232	1·1 [0·5–2·6]	0·795
>60	19	3	5	1·0 [0·3–4·0]	0·989	2	0·3 [0·0–1·5]	0·103	0·6 [0·0–1·9]	0·110	0·7 [0·2–3·1]	0·682
Hair loss												
19-35	27	17	11	0·7 [0·3–1·5]	0·351	4	0·5 [0·2–1·4]	0·166	0·4 [0·1–1·7]	0·188	1·1 [0·4– 3·1]	0·826
36-60	42	18	28	1·5 [0·9–2·6]	0·163	4	0·3 [0·1–0·8]	**0·014**	0·1 [0·0–1·2]	0·070	0·7 [0·3– 1·9]	0·475
>60	12	1	1	2·4 [0·3–20·9]	0·421	1	0·4 [0·0–6·0]	0·495	-	-	0·6 [0·1– 4·5]	0·605
Sleeping problems												
19-35	33	6	7	1·3 [0·5–3·7]	0·643	3	1·0 [0·3–3·9]	0·981	1·2 [0·2–0·8]	0·872	0·7 [0·2– 2·2]	0·523
36-60	88	19	30	1·5 [0·9–2·6]	0·139	8	0·5 [0·2–1·1]	0·092	0·6 [0·1–2·3]	0·423	0·7 [0·3– 1·3]	0·246

>60	63	4	5	0·8 [0·2–2·5]	0·620	3	0·3 [0·1–1·1]	0·070	-	-	0·3 [0·1– 1·0]	0·060
Dizziness												
19-35	39	9	8	1·0 [0·4–2·5]	0·963	5	1·1 [0·4–3·2]	0·819	0·5 [0·1–2·3]	0·341	1·0 [0·4– 2·4]	0·945
36-60	75	20	19	0·9 [0·5–1·6]	0·737	6	0·4 [0·2–0·9]	**0·023**	0·2 [0·1–1·1]	0·070	0·6 [0·3– 1·3]	0·190
>60	38	3	3	0·6 [0·1–2·7]	0·494	1	0·1 [0·0–1·2]	0·071	-	-	0·2 [0·0– 1·3]	0·094
Persistent cough												
19-35	39	10	12	1·3 [0·6–2·9]	0·494	6	1·2 [0·5–3·2]	0·685	1·0 [0·3–3·7]	0·961	1·2 [0·5– 2·6]	0·716
36-60	61	12	10	0·8 [0·4–1·8]	0·575	9	0·9 [0.4–2·1]	0·802	0·8 [0·2–3·9]	0·770	1·1 [0·5– 2·1]	0·840
>60	34	2	4	1·2 [0·2–6·4]	0·819	5	1·0 [0·2–4·8]	0·962	0·1 [0·0–2·8]	0·202	1·0 [0·4– 2·6]	0·943
Shortness of breath												
19-35	22	10	5	0·8 [0·3–2·0]	0·583	5	1·0 [0·4–2·8]	0·974	0·5 [0·1–2·3]	0·360	1·7 [0·7– 4·3]	0·254
36-60	37	13	19	1·3 [0·7–2·7]	0·343	7	0·7 [0·3–1·6]	0·336	-	-	1·4 [0·6– 3·0]	0·428
>60	18	2	3	0·9 [1·2–5·0]	0·880	2	0·4 [0·1–2·4]	0·284	0·1 [0·0–1·8]	0·909	0·8 [0·2– 3·3]	0·738
Recovery from COVID-19												
19-35	NA	71	60	0·9 [0·7–1·1]	0·347	31	0·9 [0·7–1·1]	0·263	0·8 [0·5–1·1]	0·163	NA	NA
36-60	NA	85	92	1·0 [0·8–1·2]	0·836	67	1·0 [0·8–1·3]	0·732	0·9 [0·7–1·3]	0·611	NA	NA
>60	NA	14	38	1·4 [1·0–2·1]	0·088	75	1·7 [1·2–2·4]	**0·004**	1·7 [0·8–3·5]	0·174	NA	NA

*Adjusted for duration of follow-up and asymptomatic population, n= only participants with symptoms, NA – Not applicable

Supplementary table 2. Crude and adjusted risk ratios for post COVID symptoms among participants

			**Vaccinated vs unvaccinated (Infected individuals)**								**Two doses vaccinated + infected vs uninfected**	
			**Unadjusted analysis**						**Adjusted analysis***			
**Symptoms and age**	**Uninfected (n=2447)**	**Unvaccinated (n=317)**	**Received one dose (n=340)**			**Received two doses (n=294)**			**Received two doses (n=294)**			
	**n**	**n**	**n**	**RR (95% CI)**	**p-value**	**n**	**RR (95% CI)**	**p-value**	**RR (95% CI)**	**p-value**	**RR (95% CI)**	**p-value**
Fatigue	430	82	93	1·1 [0·8–1·4]	0·667	33	0·4 [0·3–0.6]	**0·001**	0·4 [0·2–0·7]	**0·003**	0·6 [0·5–0·9]	0·008
Headache	387	69	80	1·1 [0·8–1·4]	0·590	41	0·6 [0·5–0·9]	**0·013**	0·5 [0·3–0·9]	**0·010**	0·9 [0·7–1·2]	0·389
Weakness of limbs	199	51	57	1·0 [0·7–1·5]	0·815	20	0·4 [0·3–0·7]	**0·001**	0·4 [0·2–0·9]	**0·033**	0·8 [0·5–1·2]	0·405
Persistent muscle pain	147	24	26	1·2 [0·8–1·8]	0·465	20	0·5 [0·3–0·9]	**0·017**	0·3 [0·1–0·9]	**0·028**	0·9 [0·6–1·5]	0·835
Loss of concentration	88	33	44	1·2 [0·8–1·9]	0·315	13	0·4 [0·2–0·8]	**0·007**	0·6 [0·2–2·1]	0·408	1·2 [0·7–2·1]	0·501
Hair loss	81	36	43	1·1 [0·7–1·7]	0·612	9	0·3 [0·1–0·6]	**0·001**	0·2 [0·1–0·7]	**0·005**	0·9 [0·5– 1·8]	0·794
Sleeping problems	184	29	42	1·4 [0·9–2·1]	0·189	14	0·5 [0·3–1·0]	**0·038**	0·5 [0·2–1·6]	0·264	0·6 [0·4– 1·1]	0·087
Dizziness	152	32	30	0·9 [0·5–1·4]	0·578	12	0·4 [0·2–0·8]	**0·006**	0·3 [0·1–1·8]	**0·018**	0·7 [0·4– 1·2]	0·146
Persistent cough	134	24	26	1·0 [0·6–1·7]	0·971	20	0·9 [0·5–1·6]	0·714	0·7 [0·3–1·9]	0·483	1·2 [0·8– 1·9]	0·366
Shortness of breath	77	25	29	1·1 [0·7–1·8]	0·764	14	0·6 [0·3–1·1]	0·199	0·2 [0·1–0·9]	**0·026**	1·5 [0·9– 2·6]	0·157
Loss of taste	16	28	20	0·7 [0·4–1·2]	0·150	15	0·6 [0·3–1.1]	0·076	0·5 [0·2–1·3]	0·143	-	-
Chest pains	79	23	24	1·0 [0·6–1·7]	0·922	14	0·7 [0·3–1·3]	0·201	0·7 [0·2–2·1]	0·521	5·2 [3·7–7·2]	0·000
Pins and needles sensation	92	23	31	1·3 [0·7–2·1]	0·387	6	0·3 [0·1–0·7]	0·005	0·1 [0·0–0·7]	0·021	0·5 [0·2–1·2]	0·143
Palpitations	68	19	26	1·3 [0·7–2·3]	0·403	12	0·7 [0·3–1·4]	0·285	0·5 [0·1–2·1]	0·364	6·0 [4·2–8·5]	0·000
Depression and anxiety	62	19	19	0·9 [0·5–1·7]	0·824	17	1·0 [0·5–1·8]	0·912	0·9 [0·4–2·0]	0·824	3·1 [1·9–4·9]	0·000
Abdominal pain	132	22	24	1·0 [0·6–1·8]	0·952	8	0·4 [0·2–0·9]	0·021	0·3 [0·1–1·7]	0·185	2·7 [1·9–3·7]	0·000
Problems with balance	91	21	24	1·1 [0·6–1·9]	0·826	7	0·4 [0·2–0·8]	0·017	0·2 [0·1–1·3]	0·096	-	-
Inability to control body movement	39	18	22	1·1 [0·6–2·1]	0·672	9	0·5 [0·2–1·2]	0·123	0·5 [0·1–2·2]	0·357	7·7[5·0– 11·9]	0·000
Joint pain or swelling	93	20	22	1·0 [0·6–1·8]	0·933	5	0·3 [0·1–0·7]	0·008	0·1[0·0–0·7]	0·023	0·4 [0·2– 1·1]	0·077
Loss of smell	17	23	9	0·4 [0·2–0·8]	0·009	9	0·4 [0·2–0·9]	0·025	0·3[0·1–1·3]	0·111	7·3 [3·7–14·5]	0·000
Loss of appetite	58	17	11	0·6 [0·3–1·3]	0·182	12	0·8 [0·4–1.6]	0·458	0·4[0·1–1·2]	0·114	1·7 [0·9–3·2]	0·081
Pain on breathing	25	16	16	0·9 [0·5–1·8]	0·839	8	0·5 [0·2–1·2]	0·146	0·3[0·1–1·2]	0·152	2·7 [1·2–5·6]	0·015
Nausea and vomiting	83	19	11	0·5 [0·3–1·1]	0·096	7	0·4 [0·2–0·9]	0·034	0·1[0·0–1·3]	0·085	0·7 [0·3–1·5]	0·363
Constipation	78	14	12	0·8 [0·4–1·7]	0·561	8	0·6 [0·3–1·4]	0·266	0·7[0·2–2·9]	0·664	0·9 [0·4–1·7]	0·666
Erectile dysfunction	33	12	12	0·9 [0·4–2·0]	0·861	8	0·7 [0·3–1·7]	0·462	0·9 [0·3–2·8]	0·839	2·0 [0·9–4·3]	0·071
Diarrhoea	101	15	11	0·7 [0·3–1·5]	0·329	5	0·4 [0·1–1·0]	0·045	0·3 [0·1–1·3]	0·095	3·3 [2·3– 4·7]	0·000

Double vision	26	9	17	1·8 [0·8–3·9]	0·162	3	0·4 [0·1–1·3]	0·122	0·5 [0·4–5·2]	0·535	5·1 [2·8– 9·4]	0·000
Problems speaking or communicating	18	10	15	1·4 [0·6–3·1]	0·403	3	0·3 [0·1–1·2]	0·084	0·4 [0·0–3·5]	0·391	1·4 [0·4– 4·7]	0·598
Weight loss	25	14	6	0·4 [0·2–1·0]	0·057	8	0·6 [0·3–1·4]	0·266	0·3 [0·1–1·5]	0·152	2·7 [1·2– 5·9]	0·015
Tremor / shakiness	45	8	11	1·3 [0·5–3·1]	0·588	8	1·1 [0·4–2·8]	0·879	0·1 [0·2–7·0]	0·908	1·5 [0·7– 3·1]	0·301
Problems passing urine	34	8	6	0·7 [0·2–2·0]	0·503	2	0·3 [0·1–1.3]	0·096	-	-	3·4 [1·9–6·3]	0·000
Loss of sensation, one side of the body	16	6	7	1·1 [0·4–3·2]	0·879	2	0·4 [0·1–1·8]	0·208	0·4 [0·0–5·0]	0·495	1·0 [0·2–4·5]	0·958
Skin lumps or rashes	14	6	5	0·8 [0·2–2·5]	0·674	2	0·4 [0·1–1·8]	0·208	-	-	1·2 [0·3–5·2]	0·818
Problems swallowing or chewing	18	4	5	1·2 [0·3–4·3]	0·818	3	0·8 [0·2–3·6]	0·780	-	-	9·2 [4·9–17·2]	0·000
Blood clots in veins	0	4	5	1·2 [0·3–4·3]	0·801	3	1·0 [0·2–4·2]	0·955	-	-	-	-
Kidney problems	0	5	5	0·9 [0·3–3·2]	0·925	2	0·5 [0·1–2·6]	0·415	0·7[0·1–4·2]	0·693	-	-
Fainting / blackouts	0	2	4	1·9 [0·3–10·1]	0·470	4	2·2 [0·4–11·7]	0·373	0·4[0·0–4·3]	0·448	-	-
Seizures / fits	11	3	4	1·2 [0·3–5·5]	0·775	3	1·1 [0·2–5·3]	0·926	0·4[0·0–4·3]	0·421	2·3 [0·6– 8·1]	0·206
Heart attack & stroke	0	0	2	-	-	1	-	-	-	-	-	-

*Adjusted for duration of time from first reporting symptoms, age and symptoms at infection, NA – Not applicable
