# Supplementary material for: Association between BNT162b2 vaccination and reported incidence of post-COVID-19 symptoms: cross-sectional study 2020-21, Israel
Source: NPJ Vaccines. 2022 Aug 26;7:101. doi: 10.1038/s41541-022-00526-5 (PMC9411827; doi:10.1038/s41541-022-00526-5)
Supplement: Supplementary file 2 — REPORTING SUMMARY [file 41541_2022_526_MOESM2_ESM.pdf]

nature portfolio

Corresponding author(s): Michael Edelstein

Last updated by author(s): Jun 21, 2022

Reporting Summary

Nature Portfolio wishes to improve the reproducibility of the work that we publish. This form provides structure for consistency and transparency in reporting. For further information on Nature Portfolio policies, see our Editorial Policies and the Editorial Policy Checklist.

Statistics

For all statistical analyses, confirm that the following items are present in the figure legend, table legend, main text, or Methods section.

n/a	Confirmed	
☐	☒	The exact sample size (*n*) for each experimental group/condition, given as a discrete number and unit of measurement
☐	☒	A statement on whether measurements were taken from distinct samples or whether the same sample was measured repeatedly
☐	☒	The statistical test(s) used AND whether they are one- or two-sided<br>*Only common tests should be described solely by name; describe more complex techniques in the Methods section.*
☐	☒	A description of all covariates tested
☐	☒	A description of any assumptions or corrections, such as tests of normality and adjustment for multiple comparisons
☐	☒	A full description of the statistical parameters including central tendency (e.g. means) or other basic estimates (e.g. regression coefficient) AND variation (e.g. standard deviation) or associated estimates of uncertainty (e.g. confidence intervals)
☐	☒	For null hypothesis testing, the test statistic (e.g. *F*, *t*, *r*) with confidence intervals, effect sizes, degrees of freedom and *P* value noted<br>*Give P values as exact values whenever suitable.*
☒	☐	For Bayesian analysis, information on the choice of priors and Markov chain Monte Carlo settings
☒	☐	For hierarchical and complex designs, identification of the appropriate level for tests and full reporting of outcomes
☒	☐	Estimates of effect sizes (e.g. Cohen's *d*, Pearson's *r*), indicating how they were calculated

Our web collection on statistics for biologists contains articles on many of the points above.

Software and code

Policy information about availability of computer code

Data collection	We collected the data using an online survey tool called Alchemer.com
Data analysis	Datasets were processed using Microsoft Excel and analysed using STATA version 15.

For manuscripts utilizing custom algorithms or software that are central to the research but not yet described in published literature, software must be made available to editors and reviewers. We strongly encourage code deposition in a community repository (e.g. GitHub). See the Nature Portfolio guidelines for submitting code & software for further information.

Data

Policy information about availability of data

All manuscripts must include a data availability statement. This statement should provide the following information, where applicable:

- Accession codes, unique identifiers, or web links for publicly available datasets
- A description of any restrictions on data availability
- For clinical datasets or third party data, please ensure that the statement adheres to our policy

Minimal data set, supplementary material and statistical code are published at the Harvard Dataverse via this link: https://doi.org/10.7910/DVN/N5I10C

Human research participants

Policy information about studies involving human research participants and Sex and Gender in Research.

Reporting on sex and gender	We collected data on sex, self-reported. We present the sex distribution in our population. The vast majority of questionnaires were filled in Hebrew or Arabic, where we used the standard terminology used in questionnaires for collecting such information. There is a large evidence base around vaccine effectiveness showing no difference according to gender- as a result we do not focus specifically on gender differences in this study.
Population characteristics	In this study we collected several population characteristics described in table 1 of the manuscript. This include age, ethnicity, education level, and rural/urban- the purpose was mainly to compare different study groups at baseline. However because there is a known effect of age on effectiveness, we took this into account in the analysis and adjusted for age differences in the main analysis, as well as provided an exploratory age-stratified analysis -
Recruitment	All patients who had a PCR tests analysed at one of the participating hospitals was invited- this includes all sectors of the population in terms of age, ethnicity, socio economic status and gender. We provided the questionnaire in the 4 most common languages spoken in the country to limit recruitment bias. The most likely bias are the difficulty for very elderly individuals to complete the online questionnaire, and individuals who are severely ill and may be too unwell to fill in the questionnaire. The numbers are however likely to be small. We report the baseline characteristics of the population and provide an (exploratory) age specific analysis. However the older age band may not include the very old (the age band is 60+) and it is therefore possible that the results are not generalisable to this group.
Ethics oversight	The study was approved by the ethics committees of Ziv Medical Centre, Poriah Medical Centre and Galilee Medical Centre

Note that full information on the approval of the study protocol must also be provided in the manuscript.

Field-specific reporting

Please select the one below that is the best fit for your research. If you are not sure, read the appropriate sections before making your selection.

☒ Life sciences ☐ Behavioural & social sciences ☐ Ecological, evolutionary & environmental sciences

For a reference copy of the document with all sections, see nature.com/documents/nr-reporting-summary-flat.pdf

Life sciences study design

All studies must disclose on these points even when the disclosure is negative.

Sample size	We did not specify the sample size. this is an observational study where we aimed to include as many patients as possible. Rather we focus on statistical power and do indicate. We where sub analyses are underpowered. The underpowered analyses are clearly mentioned as such and included in supplementary files
Data exclusions	We excluded 174/3572 individuals involved in the study because they did not report their vaccination status. Since the study was about the association between vaccination and reportes symtpoms, we had no other choice.
Replication	not applicable to an observational, real-world study
Randomization	Not relevant because the study design is observational
Blinding	Not relevant because the study design is observational

Reporting for specific materials, systems and methods

We require information from authors about some types of materials, experimental systems and methods used in many studies. Here, indicate whether each material, system or method listed is relevant to your study. If you are not sure if a list item applies to your research, read the appropriate section before selecting a response.

Materials & experimental systems

n/a	Involved in the study
☒	☐ Antibodies
☒	☐ Eukaryotic cell lines
☒	☐ Palaeontology and archaeology
☒	☐ Animals and other organisms
☒	☐ Clinical data
☒	☐ Dual use research of concern

Methods

n/a	Involved in the study
☒	☐ ChIP-seq
☒	☐ Flow cytometry
☒	☐ MRI-based neuroimaging
